# Effect of different bleaching strategies on microhardness of a silorane-based composite resin

**DOI:** 10.15171/joddd.2016.034

**Published:** 2016-12-21

**Authors:** Mahmoud Bahari, Siavash Savadi Oskoee, Narmin Mohammadi, Mohammad Esmaeel Ebrahimi Chaharom, Mostafa Godrati, Ayda Savadi Oskoee

**Affiliations:** ^1^Dental and Periodontal Research Center, Tabriz of University Medical Sciences, Tabriz, Iran; ^2^Department of Operative Dentistry, Faculty of Dentistry, Tabriz of University Medical Sciences, Tabriz, Iran; ^3^Department of Endodontics, Faculty of Dentistry, Tabriz of University Medical Sciences, Tabriz, Iran

**Keywords:** Hardness, silorane composite resin, tooth bleaching agents

## Abstract

***Background.*** Dentists’ awareness of the effects of bleaching agents on the surface and mechanical properties of restorative materials is of utmost importance. Therefore, this in vitro study was undertaken to investigate the effects of different bleaching strategies on the microhardness of a silorane-based composite resin.

***Methods.*** Eighty samples of a silorane-based composite resin (measuring 4 mm in diameter and 2 mm in thickness) were prepared within acrylic molds. The samples were polished and randomly assigned to 4 groups (n=20). Group 1 (controls) were stored in distilled water for 2 weeks. The samples in group 2 underwent a bleaching procedure with 15% carbamide peroxide for two weeks two hours daily. The samples in group 3 were bleached with 35% hydrogen peroxide twice 5 days apart for 30 minutes each time. The samples in group 4 underwent a bleaching procedure with light-activated 35% hydrogen peroxide under LED light once for 40 minutes. Then the microhardness of the samples was determined using Vickers method. Data were analyzed with one-way ANOVA and post hoc Tukey tests (P < 0.05).

***Results.*** All the bleaching agents significantly decreased microhardness compared to the control group (P < 0.05). In addition, there were significant differences in microhardness between groups 2 and 4 (P = 0.001) and between groups 3 and 4 (P<0.001). However, no significant differences were detected in microhardness between groups 2 and 3 (P > 0.05).

***Conclusion.*** Bleaching agents decreased microhardness of silorane-based composite resin restorations, the magnitude of which depending on the bleaching strategy used.

## Introduction

One of the factors affecting the esthetic appearance of an individual’s smile is tooth discoloration. In the past, discolored teeth underwent aggressive treatment modalities using prosthetic procedures. Now, bleaching procedures, which do not destroy tooth structures, have been suggested to solve such problems.^1^ Bleaching procedures are classified based on whether the teeth involved are vital or non-vital and whether the procedure is carried out at home or in the dental office.^2^

Bleaching agents are mostly based on peroxide and their active ingredient is hydrogen peroxide. Carbamide peroxide is disintegrated into hydrogen peroxide and urea after application. When hydrogen peroxide reacts with dental materials and tooth structures, it is disintegrated into hydroxide radicals, water and oxygen. Hydroxide radicals react with the internal and external stains of the tooth and eliminate discoloration by an oxidation reaction.^3^

Currently the most important problem of composite resins is polymerization shrinkage, with subsequent gap formation at restoration‒tooth structure interface, resulting in microleakage, recurrent caries and postoperative hypersensitivity. Recent advances in applied research on composite resins have concentrated on the use of open-circle systems such as oxirane-based resins which are cured by visible light. Oxirane-based resins have exhibited appropriate properties such as an increase in curing depth, low shrinkage, high strength and hardness comparable to that of Bis-GMA-based dental resins. Silorane is produced as a result of the reaction of oxirane and siloxane molecules. An increase in hydrophobicity of silorane is the result of the presence of siloxane monomer and a decrease in shrinkage is due to the presence of oxirane monomer in the chemical structure of silorane.^4^

Tooth-colored materials, e.g. glass-ionomers, compomers and composite resins, are widely used in modern dentistry. However, softening of these materials after application of bleaching agents affects the clinical durability of restorations carried out with these materials. Surface hardness is an important physical property of dental materials, defined as the resistance of the material against penetration and scalloping.^2^ Although bleaching agents are widely used, studies on the effects of bleaching agents on the microhardness of dental materials have yielded contradictory results, with reports of increases or decreases in or no effects on the surface characteristics of composite resins after the use of bleaching agents.^5,6^

Basting et al investigated the effect of 10% carbamide peroxide on the microhardness of three different types of packable composite resins (Fill Magic, Alert and Definite). Fill Magic and Alert exhibited a decrease in microhardness and Definite showed an increase in microhardness.^7^

Polydorou et al studied the effect of 38% hydrogen peroxide on the microhardness of six esthetic restorative materials (hybrid composite resin, flowable composite resin, microhybrid composite resin, nanohybrid composite resin, Ormocer and porcelain), reporting that 38% hydrogen peroxide did not affect the microhardness of six above-mentioned esthetic restorative materials.^8^ In addition, in a study by Mujdeci et al, 10% carbamide peroxide and 14% hydrogen peroxide did not exert a tangible effect on the microhardness of composite resin, compomer and glass-ionomer.^2^

Considering the ever-increasing use of silorane-based composite resins and a paucity of studies on the effect of all the commonly used bleaching techniques on microhardness of such composite resins, the present study was undertaken to evaluate the effect of various tooth bleaching strategies using 15% carbamide peroxide, 35% hydrogen peroxide and light-activated 35% hydrogen peroxide on microhardness of a silorane-based composite resin.

## Methods

In the present study A3 shade of a silorane-based composite resin (Filtek Silorane, 3M ESPE, St Paul, MN, USA) was used. A pilot study was carried out to determine the sample size. The sample size was determined (n=18 in each group) using the sample size formula for comparison of two means at α=0.05 and a power of 90%. However, to increase the accuracy of the study, 20 samples were included in each group, with a total of 80 samples. Eighty silorane-based composite resin samples, measuring 2 mm in thickness and 4 mm in internal diameter, were placed inside cylindrical acrylic molds and covered with a polyester matrix bond and then with a glass slab. The samples were pressed for 30 seconds with a 500-g force in order to remove extra material and create a smooth horizontal surface. Then the glass slabs and the force were removed and the samples were light-cured at a light intensity of 1000 mW/cm^2^ using a light-curing unit (Demetron A.2, Kerr Italia, S.P.A, Scafati, Italy) for 20 seconds based on composite resin manufacturer’s instructions. Then the samples were removed from the molds and stored in distilled water at 37°C for 24 hours.^7^

All the samples were polished with 600-, 1000-, 2000-, 3000- and 5000-grit abrasive papers (Silicium Carbide, Softlex, Germany), consecutively.^9^ The samples were randomly assigned to 4 groups of 20.

**Group 1 (control):** The samples were stored in distilled water at 37°C for 2 weeks.^5^

**Group 2:** The samples were bleached with 15% carbamide peroxide (Everbrite At-home Tooth Whitening Kit, Dentamerica INC, 18688 E. San Jose Ave, City of Industry, California 91748, USA) for two hours daily for 14 days.

**Group 3:** The samples were bleached with 35% hydrogen peroxide (White Image, PAC-DENT International Inc., Walnut, CA91789, USA)‏ twice for 30 minutes each time, 5 days apart from each other.

**Group 4:** The samples underwent a bleaching procedure with light-activated 35% hydrogen peroxide (Everbrite In-office Tooth Whitening, Dentamerica, 18688 E. San Jose Ave, City of Industry, California 91748, USA) under LED irradiation with Litex 686 light-curing unit (Dentamerica Inc., City of Industry, California, USA), based on manufacturer’s instructions for 40 minutes. The samples in groups 2, 3 and 4 were cleaned and rinsed with a soft brush and distilled water to eliminate the bleaching material from the sample surfaces, each day after bleaching, after each bleaching session and after the bleaching session, respectively. It should be emphasized that all the bleaching procedures in all the groups were carried out according to the instructions provided by the manufacturers of bleaching agents. The characteristics of the materials are presented in Table 1.

**Table 1:** Characteristics of study materials

**Materials**	**Type**	**Composition**	**Manufacturer**
**Filtek Silorane**	Siloranebased low shrinkage Microhybrid composite resin	Silorane resin, initiating system: camphorquinone, iodonium salt, Electron donor, Quartz filler, Yttrium Fluoride (76% weight, 55% volume, size: 0.04-1.7mm) Stabilizers, pigments	3M ESPE, St. Paul, MN, USA
**Bleaching Agents**	Everbrite At-home Tooth Whitening Kit	15% carbamide peroxide	Dentamerica INC, 18688 E. San Jose Ave, City of Industry, California 91748, USA
	White Image	35% hydrogen peroxide	PAC-DENT International Inc., Walnut, CA91789, USA
	Everbrite In-office Tooth Whitening Kit	light-activated 35% hydrogen peroxide	Dentamerica, 18688 E. San Jose Ave, City of Industry, California 91748, USA

After bleaching procedures, surface microhardness of all the samples in all the groups was determined with a microhardness device (UHL VMHT Auto, Walter UHL Technische Mikroskopie, GmbH & Co.KG, Loherstrabe 7, D-35614A Blar, Germany) using Vickers hardness test. The device was calibrated based on manufacturer’s instructions. Vickers hardness number was measured by application of a 300-g force for 20 seconds to each sample. The measurement was carried out three times in each sample at random locations and a mean value was calculated.^10^Data were analyzed with one-way ANOVA and post hoc Tukey tests at P < 0.05.

## Results

Table 2 presents means, standard deviations and standard errors of microhardness in the study groups. The maximum and minimum microhardness were observed in the control and 35% hydrogen peroxide groups, respectively. Kolmogorov-Smirnov test was used to evaluate normal distribution of data. The results of the test showed normal distribution of data (P > 0.05); therefore, data were parametric (P = 0.24). One-way ANOVA was used to compare mean microhardness values between the four study groups at P < 0.05. The results showed significant differences between the groups (P < 0.001, F = 46.2).

**Table 2:** Vicker’s microhardness (Mean ± SD) of silorane-based composite resin after bleaching with different strategies

**Groups**	**Mean + SD**	**Maximum**	**Minimum**
**Control**	63^A^ ± 1.7	66.3	60.3
**CP 15%**	55.2^B^ ± 2.1	58.6	51.3
**HP 35 %**	52.7^B^ ± 3.3	57.3	46.3
**HP 35 % L**	59^C^ ± 4.1	68.6	53

Post hoc Tukey tests were used for two-by-two comparisons of the groups. The results showed the highest mean microhardness in the control group (P < 0.001). There were no significant differences in mean microhardness between 15% carbamide peroxide and 35% hydrogen peroxide groups (P > 0.05). The mean microhardness in the light-activated 35% hydrogen peroxide was significantly higher than that in the 15% carbamide peroxide group (P = 0.001) and significantly higher than that in the 35% hydrogen peroxide group (P < 0.001).

## Discussion

Tooth bleaching as an esthetic treatment modality has attracted the attention of dentists and patients because it is a non-invasive procedure and is relatively easy to carry out.^11^ Since more than 40% of the population have had at least one of their teeth restored,^8,12-14^ many studies have evaluated the effects of bleaching agents on restorative materials. Microhardness has drawn much attention in the literature because it is an important physical characteristic of dental materials.^15-18^ Hardness of a material has been defined as its resistance against permanent surface indentation or penetration; it depends on a material’s strength, ductility, elastic stiffness, plasticity, strain, toughness, viscoelasticity and viscosity. A material’s ability to abrade or its susceptibility to abrasion by opposing teeth, materials, or being softened by chemical agents has a role in the clinical durability of dental restorations.^19^ In addition, microhardness depends on the mechanical characteristics of composites, their degradation and stainability. A reduction in microhardness due to organic matrix erosion may enhance the roughness of restorations and may decrease their wear resistance.^20^

The most important advantage of silorane-based composite resins is their low polymerization shrinkage, which can be attributed to the new technology of siloxane-oxirane.^21,22^ Considering great advances in the use of silorane-based composite resins, the present study evaluated the effects of different bleaching strategies on the microhardness of a silorane-based composite resin. Vickers hardness test was used because it is an appropriate test to determine the hardness of brittle materials.^23^

The bleaching agents evaluated in the present study were 15% carbamide peroxide, 35% hydrogen peroxide and light-activated 35% hydrogen peroxide, which are used for at-home night-guard bleaching, office bleaching and power bleaching procedures, respectively. To represent clinically relevant bleaching systems, we followed the manufacturer’s instructions.

The results of this study showed that all the bleaching agents used in the present study resulted in a significant decrease in microhardness of silorane-based composite resin compared to the control group. This finding is consistent with those of previous studies, which reported that the microhardness of composite resin materials decreased subsequent to the application of bleaching agents.^1,12,24^ Conversely, this finding is contrary to those of other studies reporting that the use of bleaching agents had no effect on the microhardness of composite resin, compomer, and glass-ionomer cement.^2,25^ Furthermore, Mourouzis et al^26^ showed that high concentrations of hydrogen peroxide and carbamide peroxide did not result in changes in microhardness and surface roughness of composite resins, including silorane-based low-shrinkage, microfilled and nanofilled types.

Therefore, it seems there is still controversy over the effects of bleaching agents on microhardness of restorative materials and the inconsistencies in the results might be attributed to differences in bleaching protocols and agents, and the restorative materials used.^27,28^

The active ingredient of the majority of bleaching agents is hydrogen peroxide which can form several temperature-, pH-, light-, co-catalyst- and transfer metal-dependent reactive oxygen species.^29^ Hydrogen peroxide has oxidative properties and can produce HO_2_^-^ and O^-^ free radicals. HO_2_^-^ is a highly reactive free radical and can disintegrate large molecules of stains to small molecules. In addition, it is hypothesized that it binds to molecular stains in inorganic structures such as protein matrices.^30^ Free radicals finally combine to produce oxygen and water molecules. A number of ramifications of this chemical process might result in the hydrolytic degeneration of composite reins, as reported by Söderholm.^31^ Softening of composite resins due to chemical processes in the oral cavity leads to abrasion of the resin in both pressure-bearing and non-pressure-bearing areas.^32^ The mechanism mentioned above and the effects of bleaching materials on the filler‒matrix interface are probably responsible for the reduction of microhardness in this composite in the present study. The detrimental effect of hydrogen peroxide on the filler‒resin interface might give rise to filler matrix debonding after uptake of water,^33^ displacing the filler particles, as shown in SEM studies.^26,33^ This resulted in a decrease in microhardness of the nano-composite Premise (Kerr, USA) after an at-home bleaching procedure.^34^

In contrast, Kamangar et al^35^ and Shafiei and Doustfatemeh^36^ showed that high concentrations of hydrogen peroxide and carbamide peroxide did not bring about any changes in the microhardness of silorane-based low-shrinkage composite resins, whereas a substantial negative effect on microhardness of methacrylate-based composite resins was reported by them. One advantage of P90 composite resin is an increase in its hydrophobicity due to the presence of siloxane in its chemical formulation that leads to the insolubility of the material.^37^ They assumed that this is probably the reason for no significant reduction in microhardness of this composite resin after bleaching. However, according to AlQahtani, the silorane-based low-shrinkage material (P90) showed more reduction in microhardness compared with nanofilled (Z350), microhybrid (Z250) and hybrid (Valux) methacrylate-based composite materials. It was postulated that this might be due to the presence of a new ring-opening silorane resin matrix and lower filler content. This type of resin matrix might be softer than other resin matrices (Bis-GMA, UDMA, and Bis-EMA) and easily soluble by bleaching agents.^38^

Another important finding of the present study was a higher mean microhardness after bleaching with light-activated 35% hydrogen peroxide compared to that after bleaching with 15% carbamide peroxide and 35% hydrogen peroxide. Composite resin hardness is brought about by the interactions of various factors associated with resin matrix and filler particles. The final hardness depends on the composition and extent of matrix resin polymerization.^39^ The samples in group 4 underwent a bleaching procedure with LED light at a light intensity over 1000 mW/cm^2^; therefore, it is hypothesized that curing percentage of composite resins increased during bleaching under the effect of this light intensity; an increase in curing percentage results in an increase in polymerization of composite resin, which in itself increases microhardness.^40^ This phenomenon compensates a decrease in composite resin microhardness due to bleaching.

In order to simulate the clinical condition, the bleaching agents in the present study were used based on manufacturers’ instructions: 15% carbamide peroxide 2 hours daily for 2 weeks; 35% hydrogen peroxide twice for half an hour each session with an interval of 5 days; and light-activated 35% hydrogen peroxide once for 40 minutes. The protocol used does not coincide with those of studies in which bleaching agents have been used continuously for several days for simulation of the cumulative effect of bleaching agents during a period of time. In general, it should be taken into account that the total duration of the use of bleaching agents with low-concentration materials was longer than that with high-concentration materials.^41,42^

As a limitation, in the present study, it was not possible to simulate the thinning effect of saliva and biofilm on tooth surfaces. In this context, the effect of bleaching agents on microhardness of restorative materials in vitro might be different from that in the oral cavity. It has been reported that salivary components might exert an accelerating effect on disintegration of carbamide peroxide, minimizing its side effects by remineralization.^41^ Therefore, it is suggested that in future studies, the bleaching agents be diluted with saliva to further simulate the clinical situation in the oral cavity. In addition, further studies are recommended to investigate the effect of bleaching agents used in the present study on other mechanical properties of silorane-based composite resins.

Furthermore, based on the findings of the present study, it might be suggested that bleaching agents should not be used indiscriminately in the patient’s mouth, and the teeth that have extensive silorane-based restorations should not be exposed to bleaching agents. Finally, patients should be informed that the physical properties of the restoration might be affected by the bleaching procedure, and the restorations might be softened. This could potentially predispose to increased adherence of cariogenic bacteria, surface wear rate, stain absorption, and potential marginal/edge strengths of these restorations and that they may need to be replaced.

## Conclusion

Under the limitations of the present study, 15% carbamide peroxide, 35% hydrogen peroxide and light-activated 35% hydrogen peroxide decreased microhardness of a silorane-based composite resin significantly. Light-activated 35% hydrogen peroxide decreased microhardness of silorane-based composite resin less than that by 35% hydrogen peroxide and 15% carbamide peroxide. However, there were no significant differences in microhardness brought about by 15% carbamide peroxide and 35% hydrogen peroxide.

## Acknowledgments

This project was carried out by the financial support from the Deputy Dean of Research at Tabriz University of Medical Sciences. The authors thank Dr Majid Abdolrahimi for editing the English manuscript.

## Authors’ contributions

This study was planned by MB and SSO. The literature review was performed by MB, SSO, NM and MG. MEEC and MG performed the experiments. The statistical analyses and interpretation of data were carried out by MEEC. MB and MG drafted the manuscript. All the authors critically revised the manuscript for intellectual content. All the authors have read and approved the final manuscript.

## Funding

The study was supported by Dental and Periodontal Research Center at Faculty of Dentistry, Tabriz University of Medical Sciences.

## Competing interests

The authors declare no competing interests with regards to the authorship and/or publication of this article.

## Ethics approval

Not applicable.
